# Study of ways to cope with alcohol-related injuries in driving

**Published:** 2019-08

**Authors:** Nader Sharifi

**Affiliations:** ^*a*^Jahrom University of Medical Sciences, Jahrom, Iran.

## Abstract

**Background:**

Alcohol is an important risk factor for road transport injuries. Road traffic crashes attributable to alcohol, are primarily associated with this high risk hazardous drinking behavior. In this study, ways to deal with alcohol-related injuries in driving have been investigated.

**Methods:**

In this overview, 60 articles with keyword prevention, alcohol consumption, injuries, and driving were investigated in the data bases of the PubMed, Elsevier and Google Scholar.

**Results:**

Linda J Cobiac study showed Raising alcohol excise tax in this high-income country would be highly cost-effective and could lead to substantial cost-savings for society. James Damsere-Derry study showed because alcohol consumption is increasing currently with motorization, it is necessary to educate alcohol users about the number of drinks required to stay below the legal limit if they are motorists as well as other road users or to prevent long-term illnesses associated with excessive alcohol use. Cheryl J. Cherpitel study showed countries with high detrimental drinking pattern (DDP) are at higher risk of injury from most causes at a given level of consumption, while countries with low restrictiveness of alcohol policy are at higher risk of injury at lower levels of consumption and at higher risk of traffic injuries at high levels of consumption.

**Conclusions:**

To reduce alcohol-related injuries in driving, it is important to increase alcohol taxes, driver training, and alcohol-restricting policies.

**Keywords:**

Alcohol, Injuries, Traffic

